# Correlation Analysis of Community Governance and Mental Health Based on Big Data and Intelligent Computing

**DOI:** 10.3389/fpsyg.2022.930691

**Published:** 2022-06-30

**Authors:** Zhenyue Ma

**Affiliations:** College of State Governance, Southwest University, Chongqing, China

**Keywords:** big data and intelligent computing, community governance, mental health correlation analysis, awareness, governance

## Abstract

With the continuous development of the era of big data, community management plays an important role in people’s mental health. Improve people’s mental health through the use of big data to improve governance of the community. To solve the problem, sequence segmentation is used in feature extraction, histogram absolute difference calculation and K-means intelligent algorithm, to analyze the existing problems one by one. Research shows that it is possible to systematically govern the community through big data and intelligent computing, so that community residents can live a higher quality of life. In order to get rid of psychological problems, it can be solved by establishing special psychological support institutions in the community and strengthening the role of the government in community management. With the continuous development of big data, the application of big data and intelligence in community governance and mental health community is a trend. With the continuous progress of society, it also brings pressure to people, which leads to the decline of people’s mental health quality. We need to do something to improve community governance and people’s mental health.

## Introduction

With the help of big data, the internet and digitization have changed the way students live and think. BP neural network analyzes the problems and constraints of using social media to carry out mental health education in colleges and universities (Schofield, 2017). There is now a growing awareness that data science is making progress in brain and mental health research. Most acknowledge that the need to use big data approaches to address complex mental health issues is a key research focus (Faurholt-Jepsen and Ostacher, 2020). Big data and machine learning may provide predictive models for clinical practice and public health systems. Big data refers to a large number of complex measurements, big data includes in addition to genomics, other “omics,” administrative, molecular, clinical, environmental, sociodemographic and even social media information. Big data and machine learning can provide predictive models for clinical practice and public health systems. Machine learning algorithms allow for prediction and stratification of clinical outcomes at the individual level, which has health benefits compared to traditional statistical methods primarily provided by machine learning. Some of the key scientific events and analytical methods used to find the cause and treatment of disease provide a concise historical perspective. The overall goal is to understand why big data and machine learning have recently emerged as ways to define, predict, and treat disease, and how they are changing the way care is conceptualized in the health sciences (Passes et al., 2019). Due to the rapid development of information technology, informatization, technology, and big data have promoted positive changes in the field of management, making important contributions to improving management efficiency and effectiveness. In China, building a “smart city” or “smart community” means using information technology to build a big data platform and management system, enabling citizens to obtain information fairly, effectively and quickly, enjoy public services, and make the government more efficient and transparent (Feng, 2015). Due to people’s attention to blockchain big data, the rapid development of its related technologies, and its wide application in national strategic guidelines, academic exploration and life production. Analyzing the role of blockchain big data platform, this paper adopts pbft blockchain consensus algorithm and technical means and smart community management mode to provide new ideas for innovative social management, which will help to improve the overall level of social services. The experimental results show that 77% of the smart community management is still in its infancy, and the smart city community governance promotes the smart community to better serve the residents and promotes the extensive and effective promotion of the characteristic community governance model (Chen et al., 2021). The application of big data provides methods and approaches for community governance, and is an important topic of community management. Analysis, mining and integration of public security management resources can provide countermeasures and suggestions for strengthening public security governance, promoting public security foundation and social governance innovation (Xiao-Long, 2019). This paper analyzes intelligent techniques for building secure communities, collecting community-based data, and heterogeneous data from multiple sources. In order to promote the safe community construction of the big data cloud service platform, according to the exact correlation of technology, security risk monitoring and early warning technology and security risk prevention technology, the “Hadoop spark HBase” decentralized framework is adopted (Daomin et al., 2019). In China, a smart community is considered to be the epitome and basic unit or module of a smart city. In order to overcome the difficulties in the construction concept, operation mode, professional talents and other aspects of the intelligent community in my country, effective measures must be taken (Guoqing and Yi, 2015). This article from the United Nations Department of Education and Technology analyzes big data collected by organizations and board reports to find possible solutions to this problem. Obtaining Weights Using AHP, Principal Component Analysis (AHP), and Entropy Methods For example, the weight of an index is determined by subjective and objective weights. Together with the normalized data, a model is obtained and the sample countries are assessed against this index. Through the main analysis, we found that the proportion of international students has the greatest impact on the health and sustainability of higher education, and has a high correlation with QS excellent schools and UN News excellent schools. Other indicators that have a greater impact include teacher-student ratio and employment rate. We use the EHSI model to give scores and compare the scores with and without policy, and find a significant increase in scores (Xiao and Yang, 2021). Humanity has entered the era of big data. With the advancement of science and technology, big data technology has been applied in various fields. However, as an almighty technology, it is bound to have some alienation. This paper analyzes the technological alienation in terms of data accuracy, privacy, and interests from the perspectives of government, society, and enterprises, and puts forward thinking and further relevant governance measures (Zhang and Han, 2017). We see population-wide data that may have been collected multiple times, including detailed population data, which are often only available in expensive and labor-intensive surveys, but with limited cost and effort. With such a dizzying array of resources, it’s easy to overlook potential limitations when the amount of data itself seems limitless. In this short article, I look at some recent advances in electronic health data related to mental health research, highlighting some potential pitfalls. The intelligent concept is implemented into the target system, organizational structure, institutional mechanism and institutional norms of urban community management, and integrates the challenges faced by urban community management. Smart management of difficult urban communities. It can be improved from the above design, open data exchange, and ensure data security (Schofield, 2017). To improve current public health strategies in suicide prevention and mental health, governments, researchers and private companies are increasingly using information and communication technologies, especially artificial intelligence and big data. Objective: The Canadian Protocol—MHSP is a tool to guide and support professionals, users and researchers using artificial intelligence for mental health and suicide prevention. Methods: A checklist was constructed from 10 international reports on artificial intelligence and ethics and two guidelines on mental health and new technologies. Checklists were validated using two rounds of Delphi consulting. Of the original 43 items, 38 items have been retained. They cover five categories: “Description of Autonomous Intelligent Systems” (*n* = 8), “Privacy and Transparency” (*n* = 8), “Security” (*n* = 6), “Health-Related Risks” (*n* = 8), “Bias” (*n* = 8). Most users find this list to be relevant and may require a version tailored for each category of target user (Zhang, 2019). In a BBC publication (December 2018), it was reported that serious mental illness has been on the rise since the early 1990s, and that women are more likely to be affected than men, but men are more likely to commit suicide. Psychological problems usually occur in childhood or by age 24. Unlike physical health conditions in physical form, mental illness is not easily detected, and there is no standard scale or way to measure it. This has caused the government to attach great importance to psychological problems (Mrch et al., 2014). In the era of big data, how to transform the method of mental health monitoring from explicit data to implicit information, from numerical data to text data, from state data to historical data, and how to balance the effectiveness and efficiency of mental health monitoring, urgently needs to be explore. This study investigates the mental health factors of college students related to family, analyzes the survey methods and results, and proposes a direction for further exploration for the mental health monitoring of college students in the mobile Internet era (Paul et al., 2019).

Through big data and intelligent computing, community governance and community residents’ mental health can be studied and analyzed, and relevant suggestions can be put forward. Digitize community management, assist community headquarters in business decision-making and intelligent scheduling, promote projects to achieve data-driven business operations, help operations become more transparent, and complete risk monitoring. By intelligently calculating the health value of people’s mental health, trend graphs, etc., it can more intuitively observe how people’s mental health changes.

## Community Governance

### Community Mental Health Service Mechanism Is Not Perfect

The problems of community mental health services mainly focus on seven obvious aspects: First, the community’s psychological needs and service supply are insufficient; there is a lack of psychological services, and the service content is limited. Second, community psychological institutions are isolated and closed, and the cooperative operation capacity is insufficient. Third, the target audience is not clear. Fourth, pay attention to the skill level and career development of medical staff. Fifth, there is a relatively large difference in the quality of psychological services in urban communities, and there is no unified standard and evaluation system. Sixth, the social psychology needs to further strengthen the health security assessment and encouragement mechanism. Seven is a unique form of community service that must be enriched and innovative.

### Community Governance Has a Modern, Institutional and Professional Impact on Mental Health

From the perspective of administrative modernization, community psychological services, which are an important part of community governance, must also keep pace with the times, reflect the characteristics of the times, pay attention to the problems and debates of the times, solve problems, and the current psychological needs of local people, supplemented by appropriate form and content. The institutionalization of community psychological services has become an important guarantee for its reform and innovation, and it is also the basis for the emergence and long-term development of community psychological services. Take the community as a place for action, take the psychological needs of the local people as a guide for action, and serve as an institutional structure to strengthen the community. The residents’ sense of identity, cohesion and happiness are the specific goals of their activities, and the systematic and professional goals of social management are to solve social conflicts at the grassroots level, promote harmonious interpersonal relationships, improve the quality of life and maintain social stability. Industrialization and multifunctional activities. There are several gaps in the domestic situation: on the one hand, psychological services are inconsistent with residents’ ideas and cognition; second, mental health services are inconsistent with residents’ demands.

### The Combination of Mental Health and Community Management Contributes to the Development of Diverse Community Management

Since the late 19th century, sociologists have used the term “community” in the context of social change to guide people’s theoretical concepts of community and its identity. The report of the 19th National Congress of the Communist Party of my country pointed out: “Establish a social psychological service system and establish a self-confidence, self-confidence, rational, peaceful and positive social thought.” The development of community mental health services is characterized by the complexity and specialization of the community management system. Therefore, it is necessary to study how to integrate psychological knowledge into community management. There is a community mental health work in our country. One of the biggest problems is the lack of government participation, the lack of motivation for spontaneous psychological support services, the lack of influence and attraction to local residents. Abroad, it is the state-led community mental health work. The development of community psychological work in China should also increase the government’s participation in related work and promote social governance. Promote the modernization of the national administrative capacity and the government-led administrative system and extend it to relevant organizations, establish specialized psychological support institutions in the community, strengthen the role of the government in community management, and promote the development of local governments.

### The Value of Community Psychological Governance

Mental health prevents adverse behaviors, diseases, and personal and family health problems, thereby ensuring the physical and psychological health of individuals and families; community psychologists have been advocating the establishment of a positive sense of community, getting rid of individualistic problems, and making There is more and more cooperation and interaction between individuals and communities; not because of gender, economic ability, social status. Whether they are healthy or not, they should treat everyone equally; social justice mainly focuses on the health and well-being of everyone’s body and mind, diversity, and treats the residents of the community with an inclusive eye; solidarity and cooperation enable psychologists and between citizens, and between psychologists and the work process.

(1) The state of quality fluctuation can be displayed; (2) More intuitively convey information about the quality of the process; and (3) After studying the fluctuation of quality, the state of the process can be grasped, so as to determine where to concentrate efforts on quality improvement.

## Distributed Big Data Intelligent Algorithm

### Sequence Segmentation and Feature Extraction

Big data sequence segmentation is the basis of histogram attribute extraction in distributed big data intelligent storage. Added non-normal distribution and normal distribution for two sets of big data. Widely used cloud technology data should be divided into two segments and then stored in two buffers of different sizes. A short buffer can store about 23–63 large datasets, while a long buffer can store about 243 large datasets. When new big data is found, put it in the short buffer. When the short buffer is full, the first step is to transfer the data stored in the short buffer to the long buffer. When the long buffer is full, the large data in the long buffer will be deleted. When samples are collected for testing, the long and short buffer ranges are compared and the differences are assessed. Compare all important data in the long buffer and short buffer, take the average, and record as $D_{\mathrm{between}}\text{.}$ Indicates that there is a difference between long and short buffers. And it is necessary to compare any two groups of big data within a long buffer range to obtain the median of the comparison results. Marked as $D_{\mathrm{long}}\text{,}$ what is mapped is a uniform level of variance in normal scenes. The specific formula is:


(1) $$D_{\mathrm{norm}}=\mathrm{long}\left(D_{\mathrm{between}}/D_{\mathrm{long}}\right)$$

The above formula and Fisher, Similar to the linear discriminant method, abnormally distributed big data series and normally distributed big data series are considered as two different types. In the case of normal scenarios in the long buffer range and abnormal scenarios in the short buffer range, $D_{\mathrm{between}}$ it represents the inter-class difference, $D_{\mathrm{long}}$ represents the intra-class difference. The larger the class spacing, the smaller the intra-class spacing. $D_{\mathrm{norm}}$ the larger the value. The greater the likelihood that large data sequences will be put into different classes of long and short buffers. Therefore, the algorithm accordingly $D_{\mathrm{norm}}$ set thresholds and identify them as incorrect data during the thresholding process. In the above calculation method, when collecting samples, the abnormal distributed big data and the normal distributed big data are divided by comparing the difference degree of the big data sequence in the long and short buffer areas. Finish LIR oriented gradients and normalization extract attributes from the graph.

(1) The state of quality fluctuation can be displayed; (2) More intuitively convey information about the quality of the process; and (3) After studying the fluctuation of quality, the state of the process can be grasped, so as to determine where to concentrate efforts on quality improvement.

### Calculation of Absolute Difference of Histogram

In order to map changes in datasets, it is suitable to classify distributed big data, combined with separate normalization RGB histogram, LIR histogram and oriented gradient histogram, giving the absolute difference between histograms for big data.

#### Normalization RGB Histogram

Two-dimensional data set is normalized RGB space, use RGB The ratio of the three components represents the data value, and the proposed data i the three components of are $R_{i}\text{,}$ $G_{i}\text{,}$ $B_{i}\text{,}$ data i points $R_{i}$ its representation is as follows:


(2) $$\mathrm{Bi}n_{R}=R_{i}\mathrm{NunBins}/\left(R_{i}+G_{i}+B_{i}\right)$$

data i points $G_{i}\text{,}$ its representation is as follows:


(3) $$\mathrm{Bi}n_{G}=G_{i}\mathrm{NunBins}/\left(R_{i}+G_{i}+B_{i}\right)$$

data i points $B_{i}\text{,}$ its representation is as follows:


(4) $$\mathrm{Bi}n_{B}=B_{i}\mathrm{NunBins}/\left(R_{i}+G_{i}+B_{i}\right)$$

Normalized RGB Schemas are less sensitive to abnormal data perturbations, but more sensitive to changes in data content.

Its component formula is as follows:


(5) $$I=\frac{1}{3}\left(R_{i}+G_{i}+B_{i}\right)$$

(6) $$S_{i}=1-\frac{1}{3\left(R_{i}+G_{i}+B_{i}\right)}\left[\min\left(R_{i},G_{i},B_{i}\right)\right]$$

$S_{i}$ and $I_{i}$ represent the data i density and mass. Density is in turn related to the quality and state of the data, so it is possible to change the state of the data that the model tracks.

#### Orientation Gradient Acquisition of Histogram

In intelligent recognition, the most important thing is edge detection, I is the current histogram, $G_{y}$ and $G_{x}$ Horizontal and vertical turns for edge detection are big data, respectively. The rough convolution formula is as follows:


(7) $$G_{x}=\left[\begin{array}{ccc} -1 & 0 & 1\\ -2 & 0 & 2\\ -1 & 0 & 1 \end{array}\right]⊗I$$

(8) $$G_{y}=\left[\begin{array}{ccc} 1 & 2 & 1\\ 0 & 0 & 0\\ -1 & -2 & -1 \end{array}\right]⊗I_{\left(8\right)}$$

data point (x,y) the edge orientation gradient of is as follows:


(9) $$Dir\left(i,j\right)=\arctan\left(G_{y}\left(i,j\right)/G_{x}\left(i,j\right)\right)$$

Dir(i,j) size is $\left[-\frac{\pi }{2},\frac{\pi }{2}\right]$ then the data (i,j) points bin is.


(10) $$\mathrm{Bi}n_{\mathrm{GradDir}}=1/\pi \left(\begin{array}{l} Dir\left(i,j\right)\\ +\pi /2 \end{array}\right)\mathrm{NumBin}s_{\mathrm{GradDir}}$$

The directional gradient of the data is not sensitive to abnormal data dynamics and slight data density fluctuations.

#### Calculate the Absolute Difference of the Histogram

The most intuitive expression of data difference is the absolute difference of the histogram. Set up big data $l_{1}$ and $l_{2}$ The histograms of $H_{1}$ and $H_{2}$, the big data histogram is Therefore, the estimation formula of the absolute difference of the big data histogram is as follows (11) shown:


(11) $$\mathrm{Differenc}e_{1,2}=\overset{n}{\underset{i=1}{\sum }}|H_{1}\left(i\right)-H_{2}\left(i\right)|$$

Now $F_{RGB}$ represents the normalization of two kinds of big data RGB the absolute difference of the histogram,


(12) $$\begin{array}{c} F_{RGB}=\mathrm{Differenc}e_{\mathrm{normalized}\;RGB}\\ =\underset{i,j}{\sum }|H_{1\mathrm{normalized}\;RGB}\left(i,j\right)-H_{2\mathrm{normalized}\;RGB}\left(i,j\right)| \end{array}$$

$F_{LIR}$ representing two kinds of big data LIR the absolute difference of the histogram,


(13) $$\begin{array}{c} F_{LIR}=\mathrm{Differenc}e_{LIR}\\ =\underset{i,j}{\sum }|H_{1LIR}\left(i,j\right)-H_{2LIR}\left(i,j\right)| \end{array}$$

$F_{\mathrm{Grad}}$ representing two kinds of big data Grad the absolute difference of the histogram,


(14) $$\begin{array}{c} F_{\mathrm{Grad}}=\mathrm{Differenc}e_{\mathrm{Grad}}\\ =\underset{i,j}{\sum }|H_{1\mathrm{Grad}}\left(i,j\right)-H_{2\mathrm{Grad}}\left(i,j\right)| \end{array}$$

The absolute difference of the above histogram is fused into a dynamic multi-feature combination, the formula is.


(15) $$F_{\mathrm{Dynamic}}=\left\{F_{RGB},F_{LIR},F_{\mathrm{Grad}}\right\}$$

It is proposed to collect a piece of data randomly, in which the number of abnormalities is 8, which are the fluctuating target, the twisted, and the masked. The data were not log-transformed. Through the calculation, it can be seen that the absolute difference of the histogram can accurately map the changes of the big data sequence. When the distributed big data changes greatly, the corresponding values will also produce different peaks, and the peaks of the same scale data are clustered together by clustering technology. Based on these branches, large-data network image sequences are stored in off-the-shelf labels before and after the buffer to complement the classification of large scattered data.

### Based on K-means Intelligent Algorithm

#### K-means Clustering

Using random initialization or K-means (Xuan, 2015) pre-sampling process selection cluster center point $p_{1},p_{2},\cdots ,p_{k}\text{.}$Pair of samples $X_{i}$ calculate the closest center point to it $p_{i}\text{,}$ and classified as $C_{i}\text{.}$


(16) $$C_{i}=\arg\min X_{i}-{P_{j}}^{2}$$

For each class j, all compute the center of the class again, as follows:


(17) $$P_{j}=\frac{\sum_{i=1}^{m}\left\{C_{i}=j\right\}X_{i}}{\sum_{i=1}^{n}\left\{C_{i}=j\right\}}$$

The similarity of the current cluster center point and the sample data objects are calculated by the weighted size estimation method of the data gain ratio, and the representative formula is as follows:


(18) $$\begin{array}{l} d\left(i,j\right)\\ =\sqrt{\gamma_{1}{\left|x_{i1}-x_{j1}\right|}^{2}+\gamma_{2}{\left|x_{i2}-x_{j2}\right|}^{2}+\cdots +\gamma_{p}{\left|x_{ip}-x_{j}\right|}^{2}} \end{array}$$

in the formula $\gamma_{i}\left(i=1,\;2,\cdots ,\;p\right)$ represents the data gain ratio.

The distributed big data in this paper is three-dimensional. When clustering, there are a total of k kind, using the clustering criterion function is:


(19) $$E=\overset{k}{\underset{i=1}{\sum }}\underset{X\in C_{i}}{\sum }\overset{3}{\underset{j=1}{\sum }}\gamma_{j}{\left(X_{ij}-m_{ij}\right)}^{2}$$

#### K-means Clustering Distance Optimization

Hypothetical center point Center is $\left(a_{2},b_{2}\right)$, the sample points need to be calculated $\left(a_{2},b_{2}\right)$. First convert the coordinates of the data points (x, y) correlate it with its two-norm to form the form $\langle \left(x,y\right),{\left(x,y\right)}^{2}\rangle$ key-value pair. The above two points can be expressed as: $\langle \left(a_{1},b_{1}\right),\mathrm{sqrt}\left({a_{1}}^{2}+{b_{1}}^{2}\right)\rangle$ and $\langle \left(a_{2},b_{2}\right),\mathrm{sqrt}\left({a_{2}}^{2}+{b_{2}}^{2}\right)\rangle$. Bound distance is the square of the difference between the two-norms, real distance is the Euclidean distance (with squares) of the two points before. Bound distance and real distance the calculation formula is:


(20) $$\mathrm{boundDis}\tan ce={\left(\sqrt{{a_{1}}^{2}+{b_{1}}^{2}}-\sqrt{{a_{2}}^{2}+{b_{2}}^{2}}\right)}^{2}$$

(21) $$\mathrm{realDis}\tan ce={\left(a_{1}-a_{2}\right)}^{2}+{\left(b_{1}-b_{2}\right)}^{2}$$

According to the axiom of “the difference between two sides of a triangle is less than the third side,” it is obvious that the result of Formula (20) is less than or equal to the result of Formula (21) bound distance It can be precomputed and such a value is recorded for each sample or sample center. Through the scaling relationship between the two-norm difference of the samples and the Euclidean distance between the samples, the repeated calculation of the distance between the sample point and the center point can be avoided, which greatly reduces the K-means the computational cost of the clustering process.

The steps are to pre-divide the data into K groups, then randomly select K objects as the initial cluster centers, then calculate the distance between each object and each seed cluster center, and assign each object to the closest distance to it. The cluster centers and the objects assigned to them represent a cluster. Each time a sample is assigned, the cluster center of the cluster is recalculated based on the existing objects in the cluster. This process will repeat until a certain termination condition is met. Termination conditions can be that no (or a minimum number) of objects are reassigned to different clusters, no (or a minimum number) of cluster centers change again, and the sum of squared errors is locally minimized.

## Community Mental Health Status

### Basic Information of the Person Under Investigation

**Figure 1:**
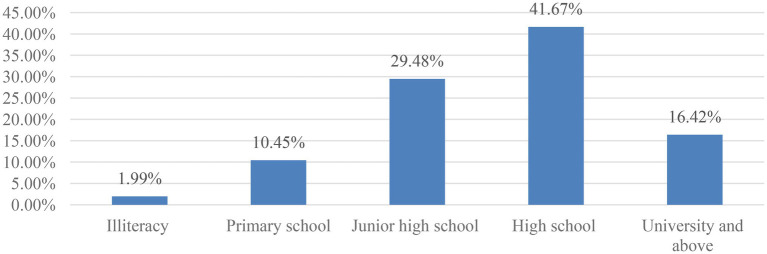
Educational level of community residents.

A total of 900 questionnaires were distributed in this survey, and 835 were recovered, of which 804 were valid questionnaires, with an effective rate of 96.29%. There are 395 males and 409 females in the sample, the age range is 18–70 years old, the average age is 42 years old; marital status, 143 people are unmarried, accounting for 17.79%, 553 people are married, accounting for 68.78%, 72 were divorced, accounting for 8.83%, 37 were widowed, accounting for 4.60%; 16 were illiterate, accounting for 1.99%, 84 were primary schools, accounting for 10.45%, and 237 were junior high school, accounting for 29.48%, there are 335 high school students, accounting for 41.67%, and 132 college students and above, accounting for 16.42%. As shown in Figure 1.

### Correlation Analysis of Intelligent Computing Mental Health

**Figure 2:**
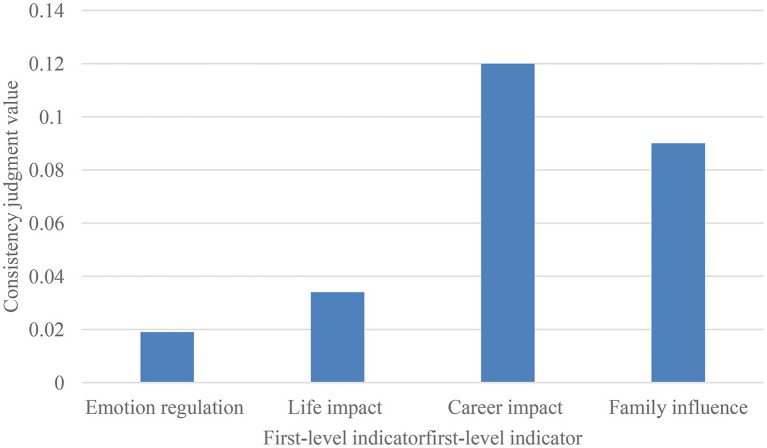
Consistency judgment results.

The statistics use the consistency judgment results of the target layer and the criterion layer in the community residents’ mental health evaluation index system. The statistical results are shown in Figure 2 below. It can be seen that the judgment value of emotion regulation is 0.019 < 0.1, which meets the requirements, and the judgment value of life impact is 0.034 < 0.1 meets the requirements, the career impact judgment value is 0.12 > 0.1 does not meet the judgment requirements, and the family impact judgment value is 0.09 < 0.1 meets the requirements. The consistent judgment results of using the model to evaluate community mental health are not all less than 0.1, indicating that the mental health indicators established by the model in this paper do not fully meet the judgment requirements and cannot all pass the consistency test.

The research result is that the mental health indicators established by the model do not all meet the judgment requirements, only the career impact judgment value is 0.12 > 0.1, which does not meet the judgment requirements; the rest of the indicators meet the judgment requirements.

**Figure 3:**
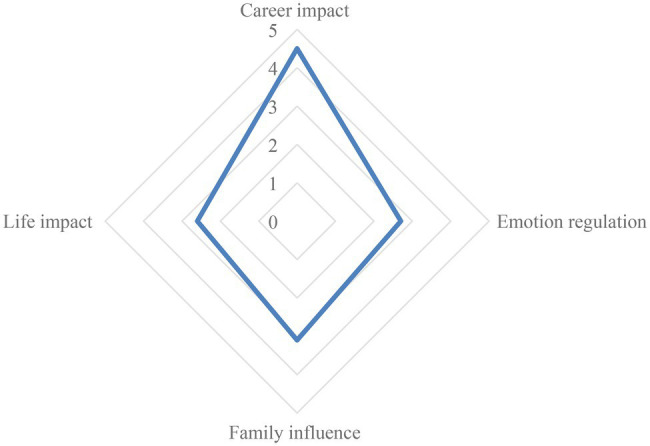
Indicator evaluation results.

Using the evaluation of the indicators obtained by the model, the total evaluation of the residents’ mental health is 3.2 points, and it can be seen that the overall mental health of the residents is relatively good. The indicator evaluation of residents is more inclined to career impact, and the evaluation scores of life adjustment and emotional impact are relatively low. When carrying out education, focus on making corresponding improvement measures for weak areas. As shown in Figure 3.

### Community Governance Under Big Data

**Figure 4:**
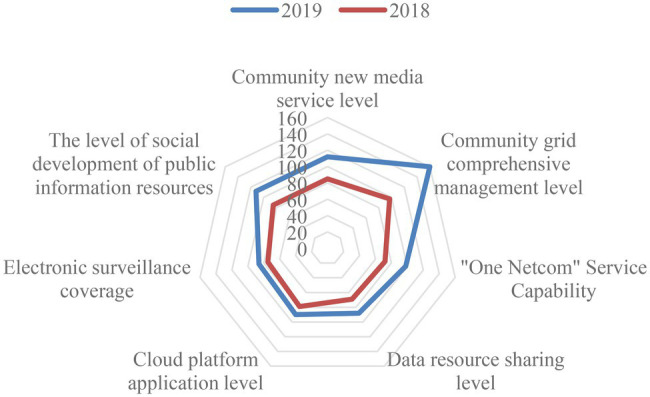
Big data community governance indicator value radar chart.

Under the governance of big data, the large-scale use of high-tech applications in the community, it can be seen from Figure 4 that the electronic coverage of the community has almost no dead ends, which ensures the safety of the community; the service level of the new media in the community has also improved significantly in the past 2 years. The information resources are disclosed through big data, so that every resident can understand the utilization of resources; the comprehensive management level of community grid is improved with the continuous maturity of information technology; For example, you can recharge water and electricity at home, which facilitates the life of residents in the community; the level of data resource sharing and the use of cloud platforms enable community residents to pay attention to the status of the community in real time.

### The Understanding of Community Residents

**Figure 5:**
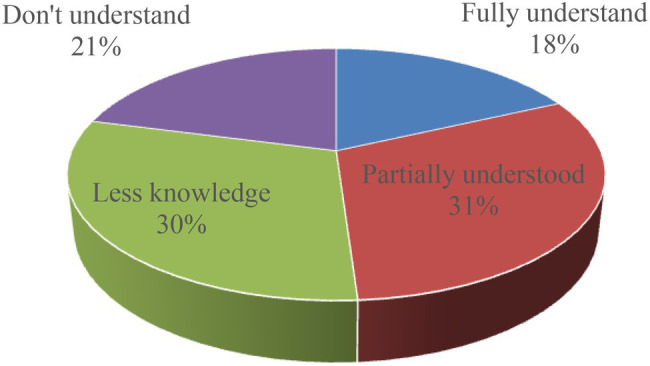
Residents’ understanding of community mental health education.

Mental health education is a grass-roots service system, and community residents’ understanding of and demand for mental health education is the focus of community mental health education. Statistics show that among the 804 people surveyed, 18.1% are “completely aware” of community mental health education work, 30.84% are “relatively familiar,” and 30.16% are “partially understand,” 30.16% of the people “understand less,” and 20.90% of the people “do not understand.” The data reflects that it can be directly seen that the residents’ understanding of mental health education is low, and professional workers who use it in the future should increase their efforts to publicize it. As shown in Figure 5.

### The Needs of Community Residents

**Figure 6:**
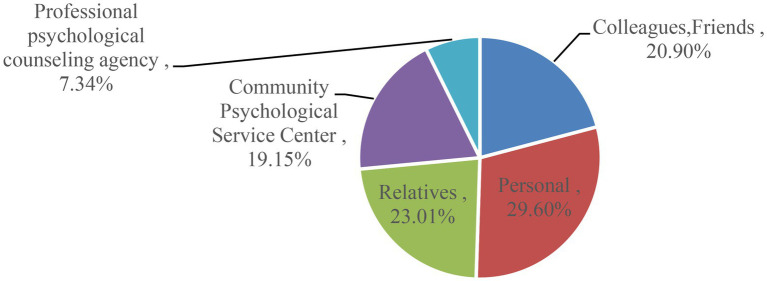
Community residents seeking help.

The survey showed that in the survey on the question “Your satisfaction with your current mental state,” 206 people were relatively satisfied with their mental health, accounting for 25.62% of the sample, and 598 people were not satisfied with their mental health, accounting for 74.38% of the sample %. This phenomenon marks the changes in social life, residents are less and less satisfied with their own mental health, and there is a potential demand for mental health services. In terms of service objects, more and more attention has been paid to mental health education in my country. Residents have different views on mental health. However, 23.01% of residents will seek help from their relatives when they have psychological problems, and 29.60% of them will seek help from their relatives 19.15% choose to seek community mental health service personnel, 20.90% choose to talk to colleagues and friends, and only 7.34% choose to go to a professional psychological counseling institution for examination. As shown in Figure 6.

### Mental Health Education in the Community

Now many communities have launched mental health education activities, using professional services to help community residents solve their daily psychological problems. The specific methods include conducting lectures on the mind, establishing psychological files, inviting professionals to conduct on-site consultation, posting relevant mental health knowledge through community advertisement boards, conducting mental health symposiums, and establishing specialized community psychological counseling institutions. Looking at the Table 1, it can be seen that the most common ways to carry out mental health education in the community are “using community publicity blue and other methods to popularize mental health knowledge” and “distributing relevant data or image materials,” accounting for 39.10% and 38.54%, respectively. The least is “hiring experts and conducting mental health lectures,” accounting for 2.03%.

**Table 1:** Ways of developing mental health education in the community.

Specific work form	Percentage (%)
Hire experts to conduct mental health talks	2.03
Hire professionals to open mental health counseling rooms	1.12
Use community bulletin boards and other methods to popularize mental health knowledge	39.10
Organizing community activities such as street-level mental health counseling	8.56
Regular one-on-one psychological counseling for specific psychological needs	7.33
Distribute relevant data or video materials	38.54
Not clear	3.32

Due to the Internet age, residents generally acquire mental health knowledge through the Internet and TV, and rarely acquire mental health-related knowledge from professional community services and newspapers and periodicals. Among them, 30% acquired knowledge from television, 35% from television, 17% from newspapers and magazines, 11% from radio, and 7% from community professional services. As shown in Table 2.

**Table 2:** Access to mental health knowledge.

Method of obtaining	Percentage (%)
Newspapers, books	17
Broadcast	11
Television	30
The internet	35
Community professional services	7

In terms of community reporting on the knowledge cycle of mental health education, 46 communities were surveyed, of which 26 communities indicated that related activities were held once a year, accounting for 56.52% of the total number of surveyed communities. Among 36.96% of the total number of surveyed communities, there are three communities that conduct monthly surveys, accounting for 6.52% of the total, and 0 communities conduct weekly surveys. As shown in Table 3.

**Table 3:** Psychological education knowledge development cycle.

Activity time	Number of communities	Percentage (%)
On average once a week	0	0.00
On average once a month	3	6.52
On average once every 6 months	17	36.96
Once a year on average	26	56.52
Total	46	100

### The Effect of Community Mental Health Education

**Figure 7:**
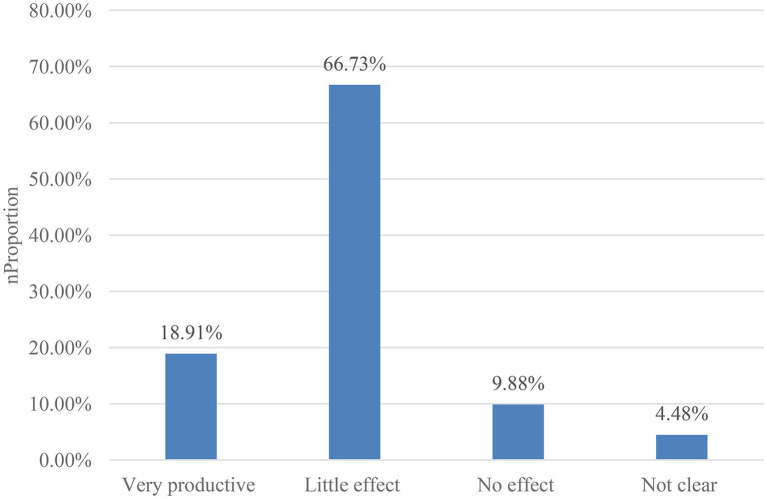
The effect of community mental health education.

Although many communities now carry out mental health education, community residents have not fully accepted it, and there is still some resistance to mental health education, but overall, community residents are still in favor of this service. The results given by 804 respondents to the questions showed that 18.91% of the people thought that health education was “very effective,” 66.73% thought it was “somewhat effective,” and 9.88% thought it was “very effective,” 4.48% thought it was “unclear.” As shown in Figure 7.

### Problems Existing in Community Mental Health Education

**Figure 8:**
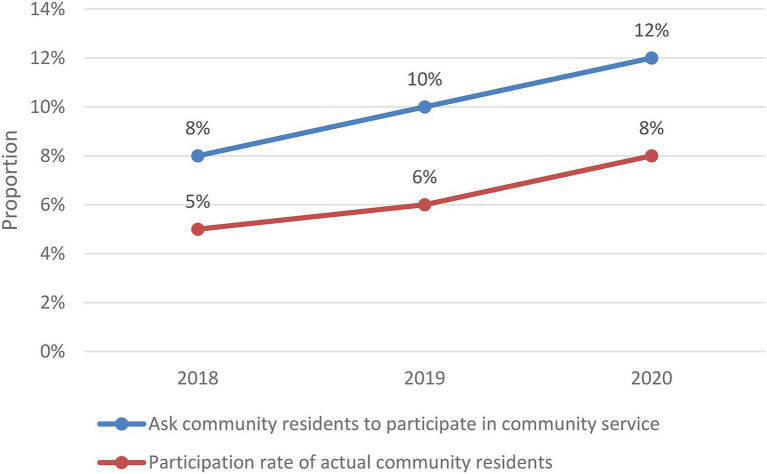
Participation of community residents.

Community residents have a low level of understanding and a low participation rate. Due to the influence of traditional ideas, existing jobs, family problems and other factors, there is still a certain misunderstanding of community health education, and it is difficult to accept this service, resulting in low participation and low understanding of community psychological education. Compared with the national standard of the national community service demonstration urban area, it is still a little far away. As can be seen from Figure 8, the progress of community psychological education is relatively slow, and the participation rate increases year by year, but it is not obvious. In order to solve this problem, it is necessary for the community to increase the publicity of mental health education and increase the participation rate of residents.

The professional degree of the staff in the mental health counseling room also determines the quality level of community mental health education. In recent years, my country has successively organized professional training for mental health education staff, and distributed a large number of psychological workers to the community. There is a certain gap compared with foreign countries in terms of professionalism and quantity. The survey shows that the main problems of our community psychological education staff are insufficient quantity and low comprehensive quality. In the development of the heart community, most of the mental health service professionals have 1–2 people, and the community with more than 3 professional qualifications only accounts for 6% of the total number of communities. In recent years, my country’s community mental health has achieved remarkable results. Among the staff surveyed in this survey, most of the workers have an education level below high school, accounting for more than 50% of the total 23% of the staff said they had the embarrassment of not knowing how to answer when a consultant asked for help. Or search online for professional help. As shown in Table 4.

**Table 4:** Educational composition of staff.

Educational level	The proportion (%)
Undergraduate and above	13
Undergraduate and above	22
High school	41
Junior high school	24.00

## Conclusion

The findings suggest that people’s need for mental health is increasing. Psychological consultation rooms can be added through community service centers, and mental health service institutions can be established to provide standardized services. Psychological training programs can be implemented by professionals in social organizations to guide and improve the behavior of individuals and social activity participants, and actively integrate into the construction of the social psychological service system and the needs of people’s lives. People are under pressure from different angles, and the need for mental health continues to strengthen and intensify. Interpersonal communication and providing mental health services “Community mental health services” and “general hospitals” that provide professional psychological counseling or psychologists, community service centers add psychological counseling rooms, establish mental health service agencies, and provide standardized services.

## Data Availability Statement

The original contributions presented in the study are included in the article/supplementary material; further inquiries can be directed to the corresponding author.

## Author Contributions

The author confirms being the sole contributor of this work and has approved it for publication.

## Funding

The work was supported by the Fundamental Research Funds for Central Universities, Southwest University (SWU1609151 and SWU1909204).

## Conflict of Interest

The author declares that the research was conducted in the absence of any commercial or financial relationships that could be construed as a potential conflict of interest.

## Publisher’s Note

All claims expressed in this article are solely those of the authors and do not necessarily represent those of their affiliated organizations, or those of the publisher, the editors and the reviewers. Any product that may be evaluated in this article, or claim that may be made by its manufacturer, is not guaranteed or endorsed by the publisher.
